# Musical playschool activities are linked to faster auditory development during preschool-age: a longitudinal ERP study

**DOI:** 10.1038/s41598-019-47467-z

**Published:** 2019-08-05

**Authors:** Vesa Putkinen, Mari Tervaniemi, Minna Huotilainen

**Affiliations:** 1 Cognitive Brain Research Unit, Institute of Behavioural Sciences, University of Helsinki, Helsinki, Finland; 2 Cicero Learning, Faculty of Educational Sciences, University of Helsinki, Helsinki, Finland; 3 Turku PET Centre, University of Turku, Turku, Finland

**Keywords:** Cortex, Human behaviour

## Abstract

The influence of musical experience on brain development has been mostly studied in school-aged children with formal musical training while little is known about the possible effects of less formal musical activities typical for preschool-aged children (e.g., before the age of seven). In the current study, we investigated whether the amount of musical group activities is reflected in the maturation of neural sound discrimination from toddler to preschool-age. Specifically, we recorded event-related potentials longitudinally (84 recordings from 33 children) in a mismatch negativity (MMN) paradigm to different musically relevant sound changes at ages 2–3, 4–5 and 6–7 years from children who attended a musical playschool throughout the follow-up period and children with shorter attendance to the same playschool. In the first group, we found a gradual positive to negative shift in the polarities of the mismatch responses while the latter group showed little evidence of age-related changes in neural sound discrimination. The current study indicates that the maturation of sound encoding indexed by the MMN may be more protracted than once thought and provides first longitudinal evidence that even quite informal musical group activities facilitate the development of neural sound discrimination during early childhood.

## Introduction

Although even newborns display some impressive auditory processing abilities, converging evidence from behavioral and brain measures indicates that the human auditory system continues to mature long after early childhood. Namely, the architecture of auditory cortical areas^1^ and the morphology of various components of the cortical auditory event-related potentials (ERPs)^2,3^ change dramatically between infancy and school-age concurrently with improvements in performance in different psychoacoustical tasks^4,5^. Music offers a particularly interesting framework for studying auditory development. Music is among the most complex stimuli the maturing auditory system encounters and therefore music processing may show a distinctive developmental trajectory. Furthermore, since adults show substantial variation in their musical skills, studying the development of music processing may provide unique insight into the emergence and determinants of individual differences in auditory processing.

The mismatch negativity (MMN) event-related potential response can be used as an index of the discrimination of musical and non-musical sounds in children. The MMN is elicited by sounds that deviate from some regular property of the preceding sounds and has been proposed to signal a failure to predict a sound on the basis prior auditory input^6^. Interestingly, some studies suggest that the MMN is a distinctly early maturing and developmentally stable index of auditory processing^7–9^. The adult MMN is a fronto-central negative response that peaks between 100 to 250 ms from the sound change onset and originates mainly from auditory cortical areas. In a seeming contradiction with the prolonged maturation of many other cortical auditory ERP components, fairly adult-like mismatch responses have been reported in young infants and even in newborns (e.g. ref.^10^). Furthermore, in contrast to the abundant evidence indicating that many other cortical auditory ERPs develop until late adolescence (e.g. ref.^2^) a number of studies have failed to find significant age-related changes in MMN amplitude across school-age^11–14^.

However, studies also imply qualitative differences between adults and young children in neural sound discrimination. Infant mismatch responses are typically later in latency and have a different scalp distribution when compared to the adult MMN^15^, and, notably, are often positive in polarity^16–19^. In fact, immature positive mismatch responses (pMMR) akin to those seen in infants have been reported still in preschool-aged children^20–23^. Some recent studies indicate that only by early school age, the mismatch response consistently attains negative polarity and thereafter increases in amplitude across pre-adolescence^24,25^. Thus, together these studies suggest that, similarly to so many other neural and behavioral indices of auditory processing, the MMN is still maturing during preschool-age. It is noteworthy, however, that the current knowledge on the development of the neural sound discrimination during pre-school age is based on relatively scarce cross-sectional evidence and direct longitudinal studies are still lacking (however, see ref.^26^ for longitudinal data on MMN development between the ages 5 to 6 years).

Until recently, developmental MMN studies have relied on rather simplistic stimuli like the odd-ball paradigm in which repeating tone is occasionally replaced by a deviant tone that differs from the standard typically in a single sound feature. Obviously, such paradigms do not resemble natural sounds such as music very closely. Thus, it remains unclear whether findings obtained in these simplistic paradigms generalize to more ecologically valid stimuli. This is an important limitation especially since some studies suggest that during preschool age, negative adult-like MMN responses can only be obtained with easily discriminable sound changes while stimuli that are complex and difficult to discriminate still elicit immature pMMRs^23,27^. Furthermore, since the maturational time course of neural sound discrimination might vary across sound features, developmental MMN studies would benefit from measuring responses to more than one type of change. Steps toward this direction have been taken by studies that have established the feasibility of examining neural sound discrimination in children with paradigms that enable rapid recording of MMN/pMMR profiles to changes in several interleaved sound regularities^28–32^.

Such paradigms have also proven sensitive to differences in neural sound discrimination between musically trained and non-trained individuals^33–36^. For instance, Putkinen *et al*.^36^ found longitudinal evidence for enhanced maturation of neural sound discrimination in musically trained children using a Melodic multi-feature paradigm that is composed of short melodies and includes changes in various musically relevant sound features. Specifically, we found that, the amplitude of the MMNs to changes in melody, rhythm, timbre and tuning increased more steeply between the ages of 9 and13 in children who had begun playing a musical instrument around the age of 7 than in children without musical training. Importantly, there were no differences in MMN amplitudes between the groups at the early stages of training, indicating that the later enhancement of the MMN in the music group did not simply reflect pre-existing group differences. Therefore, together with other recent longitudinal studies^25,37^, this study provided evidence for contribution of experience-dependent plasticity in the enhanced MMNs revealed by numerous cross-sectional studies in musically trained adults^38–40^ and children^41,42^.

The majority of studies on the neuroplastic effects of musical experience in childhood have been conducted in school-aged children with formal musical training^25,43–46^ while less attention has been devoted to the putative benefits of more informal musical activities typical for preschool-aged children. Such studies have the potential to give insight into how musical enrichment (e.g. in daycare settings) at a young age could support auditory skills in typically developing children and those with difficulties in sound processing. The available cross-sectional evidence indicates that children as young as 2–3-years who often engage in musical activities show enhanced sound processing at the cortical and subcortical levels of the auditory pathway^47,48^. Furthermore, an intervention study by Moreno, Lee, Janus, & Bialystok^49^ found that a computer-based musical training program induced changes in neural sound discrimination indexed by late discriminative negativity (LDN) in 4–6-year old children. In light of such results, it could be expected that informal group musical activities might facilitate the maturation of neural sound discrimination as indexed by the MMN/pMMR during preschool age.

To investigate whether the development of neural sound encoding indeed varies according to the amount of engagement in such musical activities, we recorded mismatch responses to different musically relevant sound changes longitudinally between the ages of 2 to 6 years in children with different levels of experience attending a musical playschool. The 45-min musical playschool sessions were held once a week and consisted of age-appropriate musical activities like singing children’s songs together and moving to music and did not involve formal training on any musical instrument. We used the same melodic multi-feature paradigm we have previously employed in school-aged children^36^. It was expected that at age of 2 years, all children would display positive polarity mismatch responses that would shift towards negative polarity with age. Furthermore, this developmental change was expected to be more pronounced in children with more extensive experience in engaging in the musical group activities. To our knowledge, this is the first longitudinal study on the maturation of neural sound discrimination in this age range and also the first to investigate whether engagement in musical playschool activities during this age period is reflected in the developmental changes in the functions indexed by the MMN/pMMR.

## Methods

### Subjects

The data were obtained in 84 recordings from 33 healthy children. The children were divided to Music and Control groups based on the duration of their attendance in a musical playschool. The children in the Music group (N = 17) had attended a playschool throughout the follow-up period from the age of approximately two years. The children in the Control group (N = 16), in contrast, had discontinued attending a playschool after the first or the second measurement. Specifically, by the time of the last measurement, the children classified as controls had attended the playschool up to two years. In contrast, the children in the Music group had attended playschool either for approximately 3 years at age 4 or approximately 5 years at age 6.

The playschool sessions were provided by an institution that specializes in training of musicians in non-classical genres such as jazz and pop. The stated aim of the playschool is to foster children’s interest towards music, to support musical learning through listening and encouraging musical interaction and expression in a supportive and positive environment. The playschool sessions consisted of joined musical activities such as singing and dancing with the guidance of a trained teacher in groups of 6–10 children. During a typical session for the 2–3-year-olds the teacher sang and played an acoustic guitar and encouraged the children to sing, play percussive instruments or move along with the music. At this age, the children were accompanied by a guardian who also participated in the activities. At ages 4–5 to 6–7, the children attended the playschool without a guardian and the activities included familiarization with musical concepts like tempo, melody and dynamics through musical play. At this age-range, the children were introduced to ‘band’ instruments such as the guitar and drums. The aim of these activities was not formal training on these instruments but rather playful familiarization with their timbres and the basic principles of how they are played (e.g. by strumming the guitar strings). The playschool sessions lasted for 45 minutes and were held on a weekly basis except for summer and winter holidays, approximately 30 times/year.

The current study is a part of a longitudinal investigation in which a new cohort of 2–3-year-old children was recruited to participate in the study every two years. The first cohorts participated in three measurements at ages 2–3, 4–5, and 6–7 (N = 18) while those recruited later participated in only two measurements at ages 2–3 and 4–5. One additional subject from the first cohort was excluded because of poor data quality due to equipment failure. For the rest of the participants the percentage of accepted trial was approximately 90% across the change types.

The respective percentage girls in the Music and Control groups were 47% and 56% for the 1^st^ and 2^nd^ measurements and 55% and 44% for the 3^rd^ measurement. The ages of the children are listed in Table 1.

**Table 1:** The ages of the children in the Music and Control groups at the times of the 1^st^, 2^nd^ and 3^rd^ measurement.

	Measurement 1		Measurement 2		Measurement 3	
	Years/Months	Std(Months)	Years/Months	Std(Months)	Years/Months	Std(Months)
Music	2/9	4	4/9	4	5/10	5
Control	2/9	3	4/10	3	5/11	3

There was no statistically significant difference between the groups in socioeconomic status (SES) as indexed by a composite score for parental income and education (Music vs. Control: t(31) = 0.240, p > 0.81) which were both measured on a 6-step scale (Income: 1 = under a 1 000 Euros/month, 6 = over 5 000/month; Education scale: 1 = comprehensive school, 2 = upper secondary school or vocational school, 3 = a higher degree than upper secondary school or vocational school which is not a bachelor’s, master’s, licenciate, or doctoral degree, 4 = Bachelor’s degree or equivalent, 5 = Master’s degree or equivalent, 6 = licenciate or doctoral level degree).

### Procedure

After the nature of the experiment and the rights of the participants were explained to the parents and children, a written consent was obtained from the parents and a verbal one from the children. The children were rewarded with movie tickets and a toy, game or a book for their participation. The experiment protocol was approved by the Ethical Committee of the former Department of Psychology, University of Helsinki, Finland. The experiment was conducted according to the committee’s guidelines and those of Helsinki declaration.

During the experiment, the children sat in a recliner chair in an acoustically attenuated and electrically shielded room. At ages 2–3 and 4–5, they were accompanied by a parent throughout the experiment. The children and the parents were instructed to concentrate on a self-selected children’s video (with the volume turned off) or a book and to remain still and avoid talking during the experiment. In general, the children were able to comply with the instructions adequately. The children were video- and audio-monitored throughout the experiment.

The duration of the current experiment was approximately 13 min. The experimental session included another auditory ERP experiment of ~50-minute duration. The order of the experiments was counterbalanced across subject. For those subjects who participated in the other experiment first, there was a ~10-min break between the current experiment and the preceding one during which the children were given juice and a snack. After the children and the parents indicated that they were ready for experimental session to be continued, they were reminded of the short duration of the second experiment compared to the first one and the children were complemented for their performance. The instructions given at the beginning of the experimental session were repeated before the current experiment was run.

### Stimuli

We employed the Melodic multi-feature paradigm that has been used in previous studies in school-aged children^36^ and in adults^35^. The paradigm is composed of 360 piano-melodies. The fundamental frequencies of the tones used to construct the melodies were between 233.1 to 466.2 Hz. Each melody consisted of a 300-ms major triad chord (first inversion) followed by two 125-ms tones (*short inter*-*tones*) and two 300-ms tones (*long inter*-*tones*) in varying order and a 575-ms tone at the end of the melody (*end tone*). Each of the Inter-tones was either the second, third, fourth or the fifth tone of the major scale while the end tone was always the tonic. The inter-stimulus interval for the chord and the inter tones was 50 ms and the silent interval between the end tone and the beginning of the next melody was 125 ms. Therefore, the duration of each melody (including the silences) was 2.1 seconds.

The paradigm included the following changes types. 1) *The melody modulation* (N = 80): One of the long inter-tones was replaced with another in-key tone. 2) *The Rhythm modulation* (N = 72): The rhythmic pattern was modulated by switching the durations of two inter-tones. 3) *The Transposition* (N = 96): The melody was transposed up or down by one semitone. 4) *Timbre deviant* (N = 96): A long inter-tone or the final tone was played with a flute timbre. 5) *Mistuning* (N = 72): A long inter-tone was mistuned by a ½ semitone. The paradigm also included a change where an inter-tone or the final tone was presented too late by a 100 ms but the responses to this change type were not analyzed because in children the prolongation of the ISI appears to introduce an artifact that makes the interpretation of response difficult (see Putkinen *et al*., 2014 for a discussion).

The Melody and Rhythm modulations and the Transpositions were presented in a roving standard manner so that after these changes the resulting new melody was repeated until the next corresponding change (on average 3–4 times). Thus, the paradigm requires that the representation of the melodic pattern is updated every few repetitions of the pattern.

The responses elicited by the changes were compared to responses elicited by standard tones that were matched with regard to duration and position within the melody. Namely, for the Melody modulation and Mistuning, the standards were the long inter-tones. For the Transpositions, the standards were the chords at the beginning of the non-transposed melodies. For the Rhythm modulations, the standards included both the long and short inter-tones. For the Timbre deviants, the long inter-tones and the final tones served as the standards. Furthermore, the standards were matched separately for each change category by the duration and type of the preceding sound in order to minimize the contribution of responses elicited by the preceding sounds to the change-standard difference.

### EEG recording

The EEG recordings were conducted either with a Neuroscan or a BioSemi Active-Two system. The number of the recordings conducted with the Neuroscan system at the age of 2, 4, and 6 were 33 out of 33, 10 out of 33, and 8 out of 18, respectively. With the Neuroscan system, the EEG (filter bandwith: 0.10–70 Hz; sampling rate: 500 Hz) was recorded using Ag/AgCl electrodes from the channels F3, F4, C3, C4 (according to the International 10–20 system), and the left and right mastoids with a common reference electrode at the nose tip. With the BioSemi system, the EEG (DC-102 Hz, sampling rate: 512 Hz, resampled to 500 Hz offline) was recorded with 32 Biosemi active electrodes on a standard Biosemi electrode cap with additional active electrodes at the right and left mastoid and nose tip. For all recordings, the electro-oculogram (EOG) was recorded with electrodes below and at the outer canthus of the right eye.

### Data analysis

The continuous EEG was re-referenced off-line to the average of the mastoid channels, filtered (0.5–30 Hz bandwidth), and divided into 400-ms epochs (from −50 ms before to 350 ms after stimulus onset). Epochs with voltage changes exceeding ± 100 μV were excluded and the remaining ones of were averaged for each change type and standard.

Mean amplitudes for each change type were calculated from the change-minus-standard difference signals over the 50-ms time windows centered on the following latencies: 250 ms for the Melody change, 300 ms for the Rhythm change, Transposition and the Mistuning and 200 ms for the Timbre deviant. Different windows were chosen for the different change types since it is known that the latencies of mismatch responses can wary depending on the change type (e.g. Pakarinen *et al*. 2007). Since the current paradigm has not been previously used in this age range we had no a priori expectations on the exact latencies of the responses and took the following ad hoc approach for selecting these time windows. Firstly, the same time windows were used for all ages for the sake of simplicity. Secondly, the windows were chosen so that they were centered approximately at the peak latencies of the negative responses at age 6 and captured the age-related positive to negative shift in both groups (if present). In accordance with the typically central distribution of the MMN in children, the statistical analyses were performed using amplitudes averaged over the central electrodes C3 and C4 for most change types (the averaging over the electrodes was done to reduce noise). For the Rhythm change, however, the age-related changes in responses appeared more pronounced over the frontal electrodes than the central ones and therefore, the statistical analysis was performed using the amplitudes of the average of F3 and F4. Note, that it is a common finding that mismatch responses to different change types differ in their scalp distribution (e.g. ref.^50^).

To identify latencies where the mismatch responses recorded at age of 2 years were significant, the responses to each change and the corresponding standard were compared with nonparametric permutation tests for paired samples in BESA statistics 1.0 software (1000 permutations, a cluster alpha level of p < 0.05). To improve the signal to noise ratio we averaged the responses across the four electrodes and pooled them across the two groups for this analysis. Note, that unlike a point-by-point testing this method controls for the Type I error^50^.

Age and group-related changes in the response amplitudes were investigated separately for each change type using linear mixed model analysis in SPSS with Age in months as a continuous predictor and Group (Music vs. Control) as a discrete predictor. Age was centered to the mean age at the time of the 3^rd^ measurement (~82 months) by subtracting this value from the ages of the subjects. Therefore, the intercept in the model (i.e. where age equals zero) was set to this age and a significant main effect of Group indicates a group difference in predicted response amplitudes at the age of approximately 82 months. Measurement (1, 2 and 3) was entered as a repeated factor to account for the correlations between amplitudes across measurements. Compound symmetry was chosen as the covariance structure on the basis of Schwarz’s Bayesian Criterion.

## Results

### Mismatch responses at age 2–3

**Figure 1:**
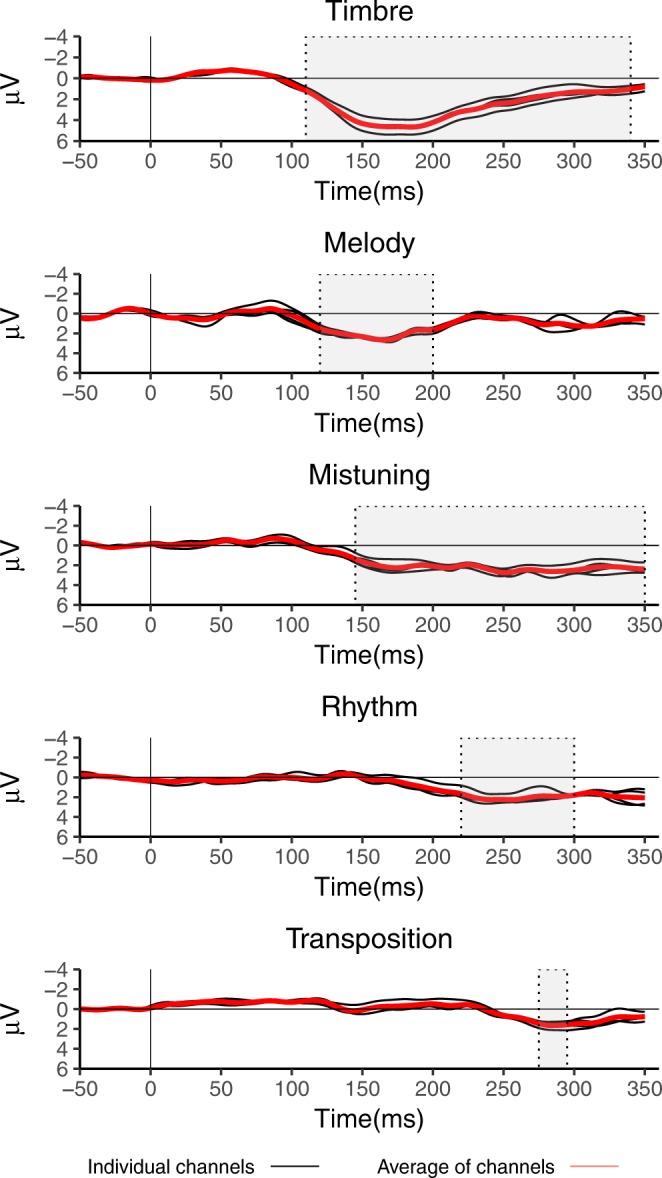
Grand average difference signals for all change types at all four electrodes (black lines) and the average of the four electrodes (red lines). The shaded areas indicate time windows where the pMMR was significantly different from zero.

Figure 1 illustrates the grand average deviant-standard responses from the first recording at the age of 2 years and the latency ranges of where the responses significantly differed from zero. Namely, the mean amplitudes of the deviant-standard difference for the Timbre, Melody, Mistuning, Rhythm and Transposition differed significantly from zero at latencies 114–340, 118–200, 146–348, 214–308, and 274–296 ms, respectively. For all change types, the significant differences were positive in polarity. Thus, at age 2–3 years the children displayed only pMMRs while no evidence for negative MMN-like responses were found for any of the change types.

### Maturation of the responses

**Figure 2:**
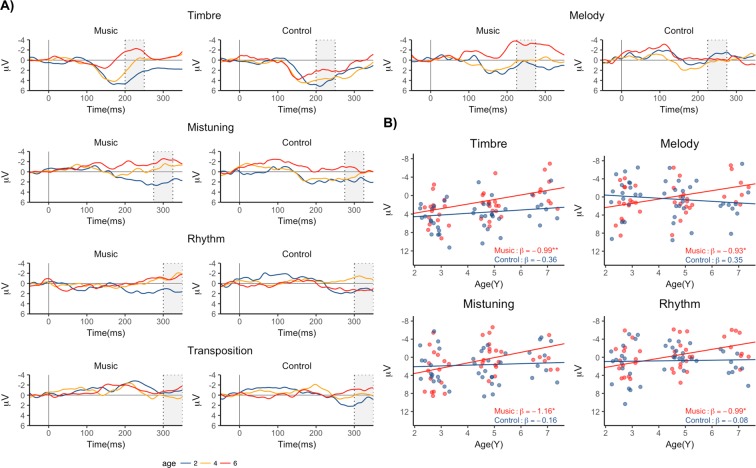
(**A**) Grand average difference signals at ages 2–3, 4–5 and 6–7 separately for the Music and Control groups. The shaded areas indicate the time windows used for calculating the response mean amplitudes. (**B**) Scatterplots illustrating the change in MMR amplitude with age in the conditions with significant group effects.

Figure 2A illustrates the deviant-minus-standard differences at the three measurements for Music and Control groups (see Supplementary Figure S1 for the responses to the deviants and standards). Figure 2B illustrates the age-related change in the response amplitudes for the four change types for which a significant group differences were obtained (see below). At age 2, both groups showed positive mismatch responses. For all changes types the responses shifted towards negative polarity with age but this shift was mainly evident only in the Music group. Specifically, the children in the Music group showed more rapid development of the neural discrimination of the changes in melody, rhythm, timbre and tuning than the children in the Control group. The results of the statistical analyses are described in more detail in the text below.

### Melody modulation

The response to the Melody modulation was significantly larger at age 6 years in the Music group than in the Control group (Main effect of Group: F(1,80) = 4.48, *p* < 0.05) but not at age 2 years (t(31) = 1.29, *p* = 0.21). The response shifted towards negative polarity with age more rapidly in the Music group than in the Control group (Age × Group interaction: F(1,80) = 5.10, *p* < 0.05). There was a significant change in response amplitude with age in the Music groups (slope: −0.93, *p* < 0.05) but not in the Control group (slope: 0.35, *p* = 0.39).

### Timbre deviant

The response to the Timbre deviant became more negative in amplitude with age (Main effect of Age: *p* < 0.001) and was larger in the Music group at age 6 (Main effect of Group: *p* < 0.05). There was no group difference at age 2 in the response amplitude (Music vs. Control t(31) = −1.10, *p* > 0.28). The age-related change in response amplitude reached significance both in the Music group (slope: −0.99, *p* < 0.01) as well as in the Control group (slope: −0.36, *p* = 0.209).

### Mistuning

The response to the Mistuning became more negative with age (Main effect of Age: *p* < 0.05) and was larger in the Music group at age 6 years (Main effect of Group: *p* < 0.05). Although the Age × Group interaction did not reach significance, there was no group difference at age 2 (Music vs. Control t(31) = 1.01, *p* > 0.32) and the examination of the age-related change in response amplitudes in the two groups indicated that the shift towards negative polarity reached significance in the Music group (slope: −1.16, *p* < 0.001) but not in the Control group (slope: −0.16, *p* = 0.643).

### Rhythm modulation

Also for the rhythm modulation, the Music group showed larger responses that the Control group (Main effect of Group, *p* < 0.05). There was also a trend for a more rapid shift towards negative polarity in the Music than in the Control group (Age × Group interaction: *p* = 0.09). Indeed, a significant change in response amplitude with age was found for the Music groups (slope: −0.99, *p* < 0.05) but not for the Control group (slope: −0.08, *p* = 0.846).

### Transposition

For the transposition, the responses appeared to shift towards negative polarity with age but the main effect of Age remained at a trend level (*p* = 0.085). No group differences were found (Main effect of Group: *p* = 0.473, Age × Group interaction: *p* = 0.125).

## Discussion

To our knowledge, this is the first longitudinal study that examines the maturation of neural sound discrimination as indexed by the mismatch response (MMN/p-MMR) from toddler age until late preschool age. Our results indicate that the neural discrimination of complex, musically relevant sound changes is still maturing during late preschool age. Specifically, we found that at age 2, children showed immature pMMRs to changes in melody, rhythm, tuning, and timbre that had shifted towards a negative MMN-like response by age 6. Furthermore, these age-related changes were mainly evident only in children who had attended a musical playschool throughout the follow-up period. The implications of these results on the typical maturation of auditory processing and the debate on the effects of musical experience on brain development are discussed below.

A number of cross-sectional studies have reported mismatch responses akin to the adult MMN already in infants even for changes whose detection requires the encoding of relatively complex auditory information such the pitch of missing fundamental^51^, polyphonic melodies^52^ and musical beat^53^. These and other such findings suggest that the MMN system operates essentially at adult level already in early childhood^15,54^. In contrast, the current results indicate that the processing of violations in complex, musical sound regularities remains immature long after infancy. Namely, all children showed positive mismatch responses at age two to most deviant types in line with previous cross-sectional evidence that positive mismatch responses can be obtained not only from infants but also from older children^21^. Furthermore, the children in the Control group did not display negative MMNs for the majority of the change types even at age six while the negative polarity mismatch responses that emerged in the Music group had a small amplitude and late latency compared to MMNs obtained in adults in the same paradigm^34,35^. Interestingly, Putkinen *et al*.^36^ found that for musically nontrained children the MMNs obtained in the Melodic multi-feature paradigm remain small or absent even at age 13. Adult non-musicians, in contrast, show robust MMNs for all change types in the paradigm^34,35^. The latter findings rule out the explanation that the absence of negative MMN-like responses in the Control group was simply due to the paradigm being too demanding for musically nontrained individuals irrespective of age. Instead, together these studies indicate that the neural discrimination of complex sound regularities that are typical for natural music develops slowly over preschool and school age and reaches adult-levels some time after pre-adolescence (see also ref.^25^).

Relative to the traditional oddball paradigms that have predominantly been used in studies reporting adult-like MMNs in young children, the Melodic multi-feature paradigm presents some additional challenges for neural sound discrimination. One key difference is that the current paradigm requires frequent updating and simultaneous maintenance of multiple regularity representations. With regard to the latter, adults tend to show similar MMNs in multi-feature and the oddball paradigms^55^ whereas children may not consistently show MMNs when the stimulus sequence includes deviants in multiple features^14^. It is noteworthy, however, that in the current study the children appeared to neurally discriminate all the change types already at age 2 as indexed by the pMMR even though they did not show adult-like MMN responses.

The current electrophysiological results are in line with behavioral and anatomical evidence suggesting protracted maturation of the auditory system. Behavioral discrimination of even basic, low-level sound properties like frequency^4,5^, intensity^4,56^ and duration^57^ is still maturing in preschool or early school-age and may even show improvement with age as late as in early adolescence^5^. Interestingly, histological studies indicate that the auditory cortex where the major generators of the adult MMN reside^58,59^ undergoes over a decade long process of axonal, neuronal and synaptic maturation^1^. However, thalamocortical connections as well as synaptic density and axons in deeper layers of the auditory cortex start maturing already during early infancy which has been speculated to underlie the emergence MMN-like responses at this age^1,60,61^. Our results indicate that these early maturing aspects of the auditory cortex cannot yet meet the processing demands of the current paradigm in an adult-like manner.

In sum, the current study suggests a prolonged maturation for the neural discrimination of changes in complex musically-relevant sound regularities that extends beyond preschool age. This conclusion is in line with previous literature indicating that the behavioral sound discrimination as well as the axonal, neuronal, and synaptic structure of the auditory cortex remains immature between early childhood and preschool-age. Moreover, the results suggest that the multi-feature MMN paradigms are not only more time-efficient and, arguably, more ecologically valid than the traditional oddball paradigms but might also be more sensitive to differences in neural sound discrimination between adults and young children.

Relative to previous studies linking musical experience and auditory development, the current study is unique in terms of the combination of the age range, the type of musical activity and the neural processes examined. Firstly, the few published longitudinal studies comparing MMN maturation between musically trained and non-trained children have all been conducted in school-aged children^25,36,37^. These studies have found that musical training is associated with steeper increase in MMN amplitude with age for various features of musical as well as speech sounds. The current results extend these findings by suggesting that the ‘musician’ advantage in the maturation of neural sound discrimination can arise already during preschool-age and does not require formal training on a musical instrument. Previous longitudinal and cross-sectional ERP studies in preschoolers, in turn, have linked taking music lesson with enhanced brainstem encoding of syllables and cortical processing of piano and violin tones as indexed by the auditory brainstem^48^ and cortical responses^62^. However, these studies only looked at the processing of single, repeated stimuli whereas the current paradigm enabled us to examine more complex auditory processing that requires parallel maintenance and monitoring of multiple complex sound regularities. The current study established that already preschoolers have the capacity for such demanding auditory processing even when they are not explicitly trying to discriminate the sounds, and further, that musically more experienced children did this in a qualitatively different way relative to children with less musical exposure.

Since most studies examining the neuroplastic effects of musical training are cross-sectional and the many longitudinal studies on the issue do not include random assignment, the literature could be criticized as being mostly inconclusive with the contribution of experience vs. innate predispositions. Due to the quasi-experimental design, the current study cannot unequivocally establish causality between the experience in playschool and enhanced development of sound features. Indeed, studies in monozygotic and dizygotic twins indicate heritability of the MMN amplitude^63^ and some musically relevant perceptual and motors skills^64–66^. Furthermore, personality factors, that may have a strong genetic basis, predict whether an individual seeks out and persists in musical training and thereby indirectly influence music perceptual skills^67^.

Importantly, however, we found no significant differences in mismatch response amplitudes between the groups in the baseline measurement at age two. Thus, the group differences found at age six did not simply reflect initial differences in neural sound discrimination between children (cf. refs^68,69^). One could, of course, argue that children whose auditory development is faster for genetic reasons more likely to continue in a musical playschool. Although this explanation cannot be completely ruled out, it seems implausible that informal and inclusive musical playschool activities would be perceptually so demanding as to deter children without genetic predisposition for high-level auditory skills. It also bears mentioning that the reasons the parents reported for not enrolling their children to the playschool were mainly related to scheduling issues and not their children’s perceived lack of musical aptitude or engagement in musical activities. As mentioned above, longitudinal studies have found compelling evidence that different indices of brain function including the MMN are modified by musical activities^37,70^. Furthermore, laboratory studies have unequivocally demonstrated that even short-term training enhances cortical sound processing^71,72^. It stands to reason that motivating real-life musical activities should have similar if not stronger effects. Moreover, both behavioral and neural measures in adults and children indicate that incidental everyday musical exposure can is sufficient to fine-tune auditory processing to the idiosyncratic features of one’s musical culture^73,74^. Importantly for the current context, some aspects of such musical enculturation appear to be accelerated in young children by interactive musical experience in playschool settings^75,76^.

In sum, the main finding of the current study was that neural processing of melody, rhythm, timbre and tuning matured faster in children attended a musical playschool throughout their early childhood compared to children with less musical experience. Future studies could assess whether these benefits extend to the processing of non-musical sounds such as phonemes (for behavioral evidence in line with this notion see ref.^77^) and examine the long-term effects of early informal musical activities on later auditory skills and motivation towards musical engagement. In conclusion, the current results indicate that age-appropriate musical group activities benefit the maturation of complex sound processing in early childhood. These results add to the evidence that informal musical experience facilitates auditory skill development.

## Supplementary information

Supplemantary figure

## Data Availability

The data obtained in the current study are available from the corresponding author on reasonable request.
