# Supplementary material for: Musical playschool activities are linked to faster auditory development during preschool-age: a longitudinal ERP study
Source: Sci Rep. 2019 Aug 5;9:11310. doi: 10.1038/s41598-019-47467-z (PMC6683192; doi:10.1038/s41598-019-47467-z)

Musical playschool activities are linked to faster auditory development during preschool-age: a longitudinal ERP study.

Vesa Putkinen, Mari Tervaniemi & Minna Huotilainen

Supplementary figure F1

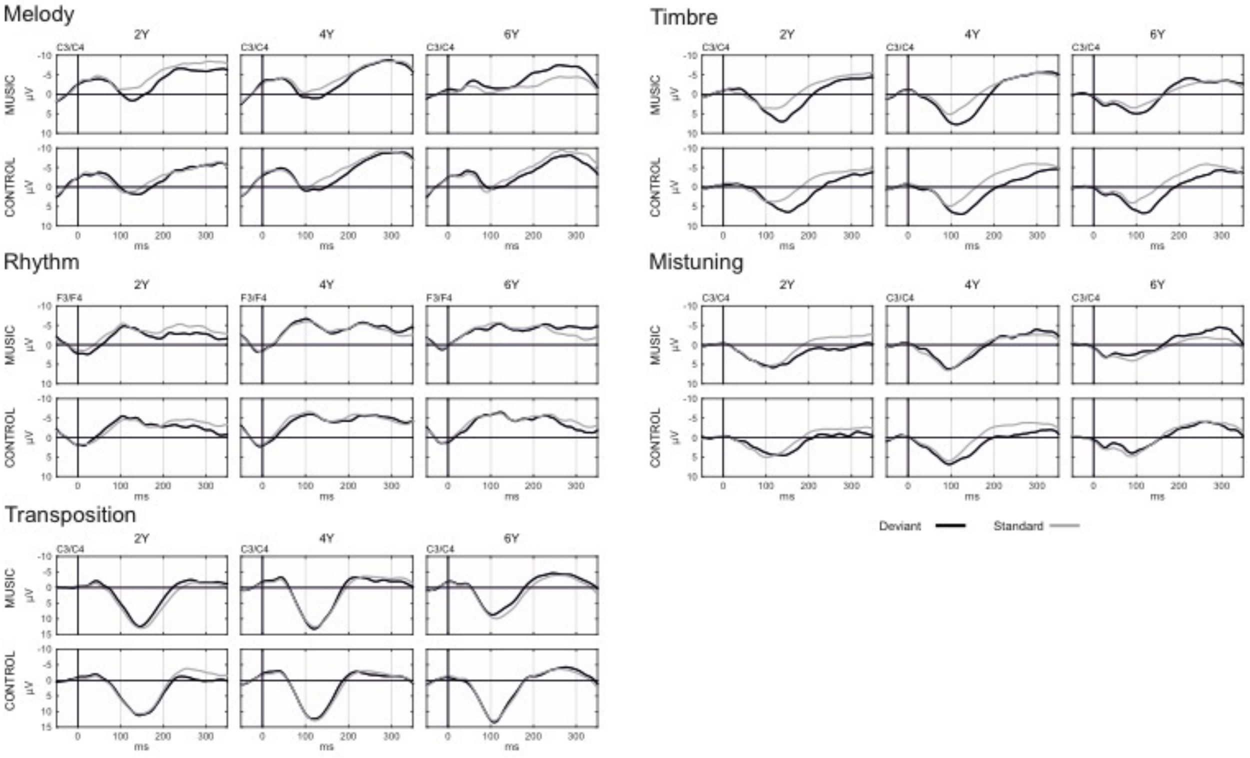

Supplement: Supplementary file 1 — Supplemantary figure [file 41598_2019_47467_MOESM1_ESM.pdf]
